# Case report of transient corneal edema after immunization with adenovirus-vectored COVID-19 vaccine

**DOI:** 10.1097/MD.0000000000030041

**Published:** 2022-07-22

**Authors:** Jae Yeon Lee, Sang Beom Han

**Affiliations:** a Department of Ophthalmology, Kangwon National University College of Medicine, Kangwon National University Hospital, Chuncheon, Korea.

**Keywords:** cornea edema, COVID-19, COVID-19 vaccine

## Abstract

To report a case of bilateral transient corneal edema presumably associated with adenovirus-vectored coronavirus disease 2019 (COVID-19) vaccination that resolved with eye drops treatment.

**Patient concerns:**

A 55-year-old Asian woman presented with sudden onset of bilateral visual disturbance developed 6 days after immunization with an adenovirus-vectored COVID-19 vaccine (AstraZeneca, London, United Kingdom). She underwent uneventful cataract surgery in right and left eyes 2 months ago and maintained good visual acuity bilaterally. Slit-lamp examination showed bilateral mild corneal edema that was confirmed with anterior segment optical coherent tomography. Anterior chamber and vitreous were clear bilaterally. Both fundi were normal.

**Diagnoses:**

The patient was diagnosed with corneal edema following adenovirus-vectored COVID-19 vaccination.

**Interventions:**

She was prescribed with prednisolone acetate 1% eye drops bilaterally.

**Outcomes:**

Treatment with topical steroid for 2 weeks resulted in resolution of the corneal edema and improvement of the visual acuity bilaterally.

**Lessons:**

This case suggests that transient corneal edema can develop following adenovirus-vectored COVID-19 vaccination. Prompt ophthalmologic evaluation and treatment may improve the corneal edema.

## 1. Introduction

With the rapid development of coronavirus disease 2019 (COVID-19) vaccines and rigorous efforts to encourage vaccination worldwide, millions of people have received COVID-19 vaccines since December 2020. Although systemic adverse effects, such as, headache, fatigue, malaise, myalgia, arthralgia, and thrombosis, have been demonstrated,^[1]^ ophthalmologic side effects have rarely been reported. We recently experienced a case of bilateral corneal edema accompanied with malaise, fatigue, and myalgia following adenovirus-vectored COVID-19 vaccination.

## 2. Case report

**Figure 1.**
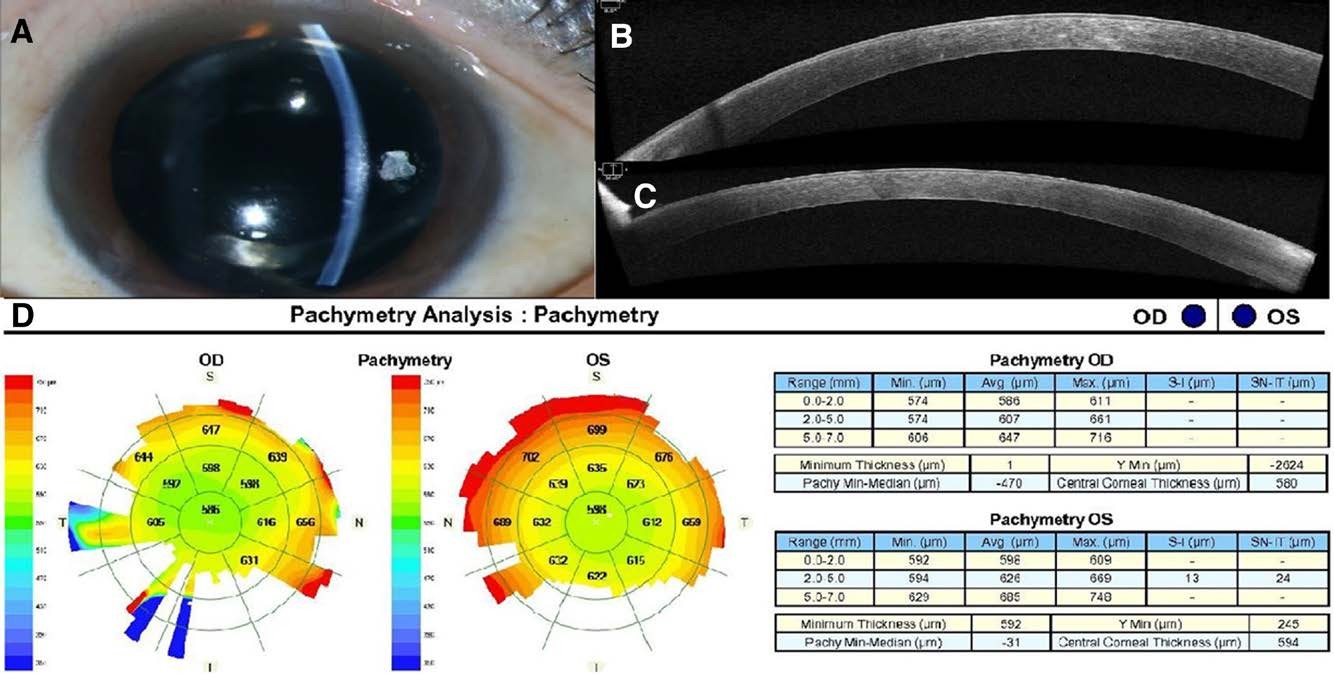
(A) Anterior segment photography shows corneal edema in the left eye. (B, C) AS-OCT demonstrates corneal edema in the right (B) and left (C) eyes, respectively. (D) Pachymetry analysis shows CCT of 580 and 594 μm in the right and left eyes, respectively. AS-OCT = anterior segment optical coherence tomography, CCT = central corneal thickness.

**Figure 2.**
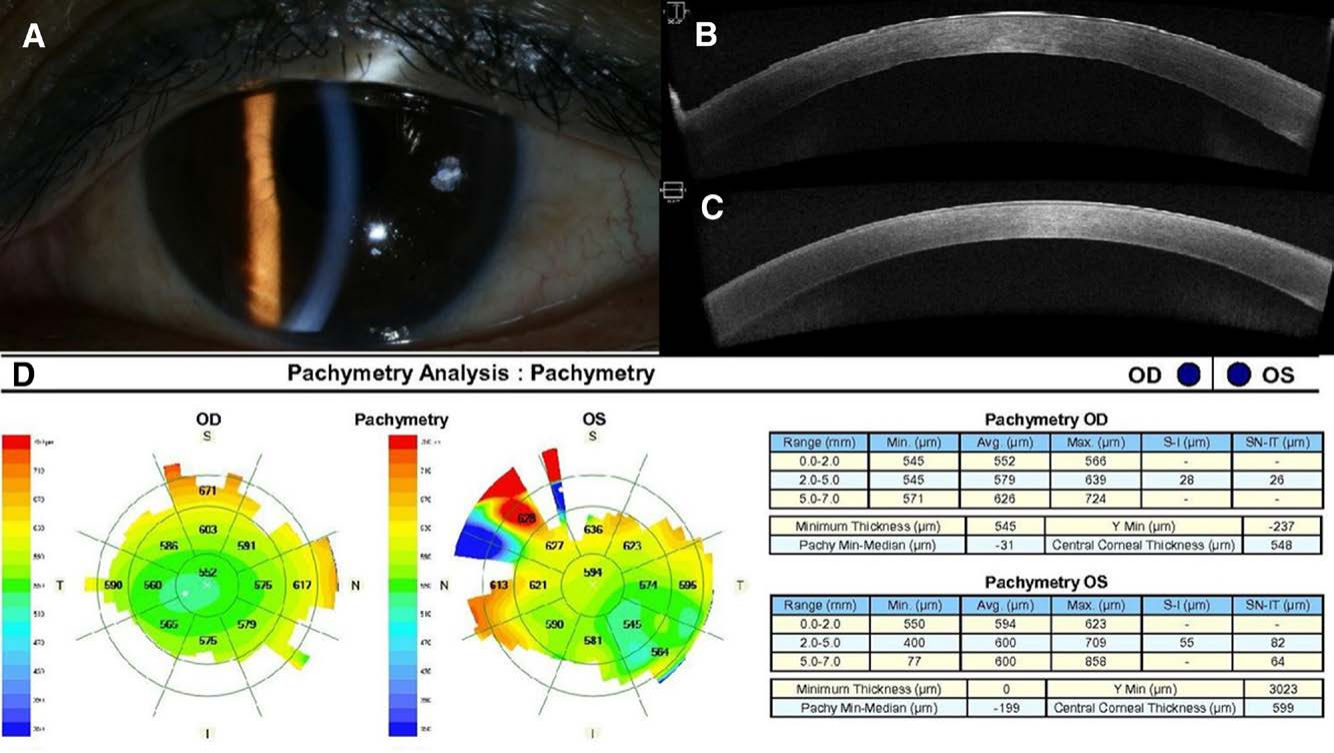
(A) Anterior segment photography shows resolution of the corneal edema in the left eye. (B, C) AS-OCT demonstrates resolution of the corneal edema in the right (B) and left (C) eyes, respectively. (D) Pachymetry analysis shows decreased CCT (548 and 599 μm in the right and left eyes, respectively). AS-OCT = anterior segment optical coherence tomography, CCT = central corneal thickness.

A 55-year-old Asian woman presented with sudden onset of bilateral visual disturbance and ocular pain. She received an adenovirus-vectored COVID-19 vaccine (AstraZeneca, London, UK) 6 days ago. She underwent uneventful cataract surgery in both eyes 2 months ago, and best corrected visual acuity (BCVA) was 20/20 bilaterally at postoperative 1 month. At presentation, BCVA was 20/30 bilaterally, and intraocular pressure was 10 mm Hg in the right eye and 10 mm Hg in the left eye. Slit-lamp examination revealed bilateral mild corneal edema (Fig. 1A). Anterior chamber and vitreous were clear bilaterally. Both fundi were normal. Endothelial cell density was 2849/mm^2^ and 2778/mm^2^ in the right and left eyes, respectively. Anterior segment optical coherence tomography (AS-OCT) demonstrated mild corneal edema (Fig. 1B, C) with a central corneal thickness of 580 and 594 μm in the right and left eyes, respectively (Fig. 1D). She was prescribed prednisolone acetate 1% eye drops (4 times a day). Two weeks later, her BCVA improved to 20/25 bilaterally, and intraocular pressure was 11 mm Hg in both eyes. Slit-lamp examination showed complete resolution of the corneal edema in the right eye and minimal focal edema at nasal side in the left eye (Fig. 2A). AS-OCT demonstrated the resolution of corneal edema (Fig. 2B, C) with central corneal thickness of 553 and 579 μm in the right and left eyes, respectively (Fig. 2D).

## 3. Discussion

In this study, the bilateral involvement, accompanying systemic symptoms, and temporal association suggest that corneal edema might develop in association with the adenovirus-vectored COVID-19 vaccine, although there is no definite evidence for a causal relationship.

Phylactou et al^[2]^ reported 2 cases of endothelial graft rejection in patients with previous Descemet’s membrane endothelial keratoplasty surgery following immunization with COVID-19 messenger RNA vaccine,^[2]^ in which the immune-mediated reaction elicited by the vaccination might exacerbate the allograft rejection.^[2]^

Corneal edema in the present case might also be associated with autoimmune response probably triggered by the immunogenicity of the vaccine, either by reinforced innate host immune response or molecular mimicry between a specific antigen in the vaccine and a self-antigen in corneal endothelial cells.^[1]^ Immune-mediated corneal inflammatory reactions, such as, immunological corneal ring and interstitial keratitis, have also been reported after varicella vaccination.^[3,4]^

There is also a possibility that adenovirus vector might be associated with corneal edema. Canine adenovirus vaccine was shown to be associated with corneal edema.^[5]^

Miyadera et al^[6]^ also reported that administration of adenovirus vectors encoding foreign transgenes to the corneal stroma resulted in transient focal corneal edema, which was resolved with topical steroid. These findings suggest that adenoviral capsid can be an immunogen that can lead to corneal edema.^[6]^ We believe further studies are needed for elucidation of the exact pathogenesis of corneal edema.

This case shows that corneal edema can develop following adenovirus-vectored COVID-19 vaccination and suggests that ophthalmologic examination is needed for patients complaining of blurred vision after COVID-19 vaccination, particularly those with a recent history of intraocular surgery. We do not believe the COVID-19 vaccination should be discouraged because of the possibility of cornea edema, as corneal edema was mild and successfully treated with topical steroids.

## Author contributions

Conceptualization: Sang Beom Han

Data curation: Jae Yeon Lee

Investigation: Jae Yeon Lee, Sang Beom Han

Methodology: Jae Yeon Lee, Sang Beom Han

Writing original draft: Jae Yeon Lee

Writing review & editing: Sang Beom Han
